# Pharmacokinetic Study of Safrole and Methyl Eugenol after Oral Administration of the Essential Oil Extracts of *Asarum* in Rats by GC-MS

**DOI:** 10.1155/2021/6699033

**Published:** 2021-03-19

**Authors:** Yuqi Fan, Dongyue Yang, Xuhua Huang, Guangzhe Yao, Wei Wang, Mengyuan Gao, Xiaohua Jia, Huizi Ouyang, Yanxu Chang, Jun He

**Affiliations:** ^1^First Teaching Hospital of Tianjin University of Traditional Chinese Medicine, 300193 Tianjin, China; ^2^State Key Laboratory of Component-Based Chinese Medicine, Tianjin University of Traditional Chinese Medicine, Tianjin 301617, China

## Abstract

*Asarum* is a traditional medicine and has been widely used as remedies for inflammatory diseases, toothache, headache, local anesthesia, and aphthous stomatitis in China, Japan, and Korea. Our previous research found that safrole and methyl eugenol were vital compounds that contribute to distinguish the different species and raw *Asarum* and its processed products apart. The pharmacokinetics of safrole and methyl eugenol after oral administration of *Asarum* extract has not been reported yet. In this study, a rapid and simple gas chromatography–mass spectroscopy (GC–MS) method that has a complete run time of only 4.5 min was developed and validated for the simultaneous determination and pharmacokinetic study of safrole and methyl eugenol in rat plasma after administration of *Asarum* extracts. The chromatographic separation was realized on a DB-17 column (30 m × 0.25 mm × 0.25 *μ*m). And detection was carried out under selected ion monitoring (SIM) mode. Plasma samples were pretreated by *n*-hexane. The pharmacokinetic parameters provided by this study will be beneficial for further developments and clinical applications of *Asarum*.

## 1. Introduction

*Asarum*, with the Chinese name Xixin, is a perennial plant that is widely distributed in China, Japan, and Korea. It originates in the roots and rhizomes of *Asarum sieboldii* Miq., *Asarum sieboldii* Miq. var. *seoulense* Nakai, and *Asarum heterotropoides* Fr. Schmidt var. *mandshuricum* (Maxim.) Kitag [1]. *Asarum* is a traditional medicine and has been widely used as remedies for inflammatory diseases, toothache, headache, local anesthesia, and aphthous stomatitis in many countries [2]. Phytochemical studies on this herb showed that the components of *Asarum* included volatile oil, flavonoids, aristolochic acids, and furofurans-type lignan [3, 4]. Among these components, volatile oils are not only the major bioactive compounds but also the toxic compounds of *Asarum*. It has attracted wide attention from researchers because of its high content and strong activities. Modern pharmacological studies have demonstrated that various volatile oil ingredients in *Asarum* have a lot of activities such as antibacterial, treatment of allergic rhinitis, antinociceptive, anti-inflammatory, and antiallergic [5–9]. On the other hand, toxicological researchers found that the volatile oil ingredients in *Asarum* have strong phytotoxic activity and it was toxic to D. *farinae* adults [10, 11].

Prior to the use of traditional Chinese medicine, most toxic raw herbs must be subjected to chemical and/or physical pretreatment processes after harvest for preservation, detoxification, or efficacy enhancement [12]. After processing, the chemical components of raw herbs and processed products often change, and these changed components might be the material basis of the herb increasing efficiency or reducing toxicity [13]. Our previous research used PLS-DA analysis to distinguish and predict *Asarum* samples of different species and its processed products, and the results indicated that the safrole and methyl eugenol were identified as key compounds that can be beneficial to differentiate the different species apart. Apart from that, both of these two volatile oils were notable index constituents to differentiate raw *Asarum* and its processed products [14].

The methyl eugenol and safrole are common volatile oil compounds found in many plants and had been widely used as a flavoring agent in people's daily life [15, 16]. But both of these two compounds were found to be carcinogenic and have been set as a group 2B carcinogen by International Agency for Research on Cancer (IARC) [17, 18]. Some researchers have studied the elimination of methyl eugenol from serum after administration of the standards of methyl eugenol [19]. However, to the best of our knowledge, there is still a lack of information on the pharmacokinetics study of methyl eugenol and safrole simultaneously in vivo after oral administration of *Asarum* essential oil extract.

Many studies have shown that these two compounds might be the main active ingredients of *Asarum* for various medicinal effects such as acaricidal activity and antifungal effect [20, 21]. Therefore, understanding the pharmacokinetic activities of these two components in vivo after administration of *Asarum* extracts is of great significance for finding out how *Asarum* exerts its pharmacological or toxicological effects in vivo.

Previously developed methods for the identification and quantitation of the bioactive components of *Asarum* include the headspace GC-MS and the UHPLC-Q-TOF/MS [22, 23]. The focus of previous researches was on the components of *Asarum* in vitro. GC/MS in the selected ion-monitoring mode (SIM) method was always used for identification and quantitation in recent years, and it is more specific compared to scan mode [24–26]. In this current study, a simple and sensitive GC-MS method performed under SIM mode was established for simultaneously quantifying safrole and methyl eugenol in rat plasma. This method has a complete run time of only 4.5 min, and it was first used for the pharmacokinetic analysis of safrole and methyl eugenol in rat plasma simultaneously after administration of *Asarum* essential oil extracts.

## 2. Material and Methods

### 2.1. Materials

**Figure 1:**
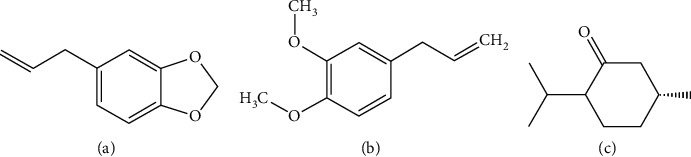
The chemical structures of (a) safrole, (b) methyl eugenol, and (c) IS.

Safrole, methyl eugenol, and menthone (IS) were obtained from Desite Biotech Co., Ltd. (Chengdu, China). The chemical structures of them are displayed in Figure 1. *Asarum* was purchased from Liaoning province in China. N-Hexane was purchased from Kangkede Technology Co., Ltd (Tianjin, China). A Mili-Q water purification system (Millipore, Milford, MA, USA) was used for preparing the ultrapure water.

### 2.2. GC-MS Analysis

The GC–MS system (Shimadzu GC-MS QP-2010 Ultrasystem) was coupled with an AOC-20i autosampler. The column used was a DB-17 capillary column (30 m × 0.25 mm × 0.25 *μ*m film thickness). 99.99% high purity Helium was used as the carrier gas with a flow rate of 1.3 mL/min. The column temperature was programmed as follows: initial temperature 90°C, 20°C/min to 106°C, 30°C/min to 136°C, 40°C/min to 210°C, and finally 5.5°C/min to 214°C. The injection volume was 1 *μ*L, and the injection temperature was 250°C.

**Figure 2:**
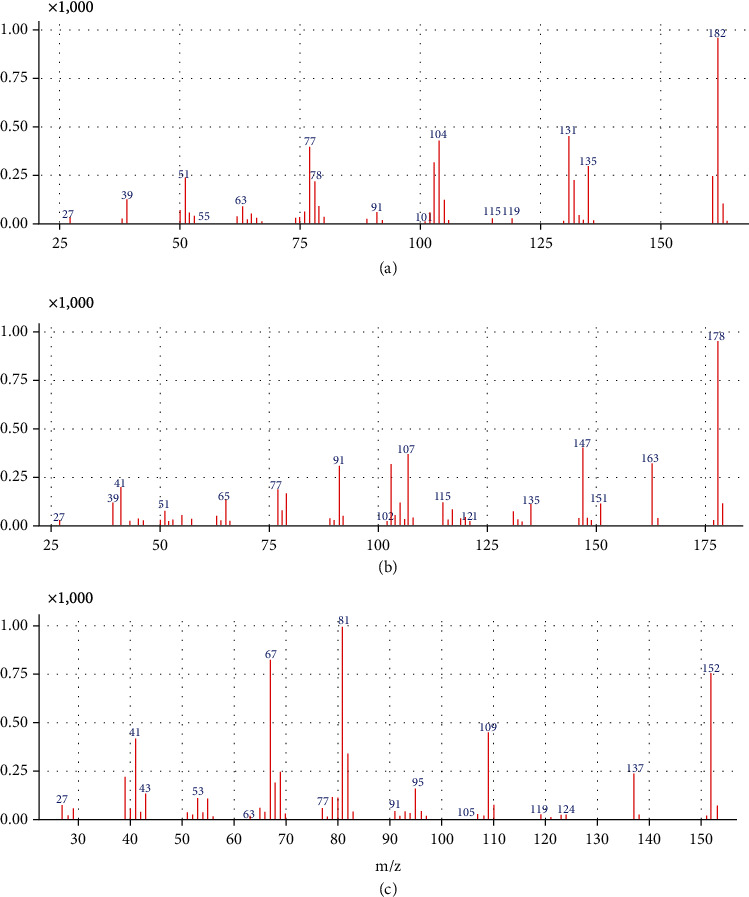
Mass spectrums of (a) safrole, (b) methyl eugenol, and (c) IS.

The MS data were acquired in the electron-impact mode, and the SIM mode was used for quantification. The three characteristic ions of safrole were 162.0, 131.0, and 104.0 and that of methyl eugenols were 178.0, 163.0, and 147.0 (ions in bold fonts were used for the quantification). The obtained mass spectrums are shown in Figure 2. The transfer line temperature was set at 250°C, and the ionization source temperature was set at 230°C. The total run time was 4.5 min with a solvent delay of 3 min.

### 2.3. *Asarum* Extracts Preparation

The *Asarum* powder sample was accurately weighed 50.0 g and extracted by an essential oil extractor for 6 h at 100 volt. After cooling to room temperature, the extracted essential oils were collected, and anhydrous sodium sulfate was then used to dehydrate the volatile oil. The obtained essential oil was stored at 4°C until analysis. The contents of methyl eugenol and safrole in the extract were 38.3 and 13.2 mg/g, respectively.

### 2.4. Preparation of Standard and Calibration Curves

Safrole, methyl eugenol, and menthone (internal standard, IS) were separately weighed 10 mg and dissolved with an appropriate volume of *n*-hexane to a concentration of 140 *μ*g/mL, 100 *μ*g/mL, and 50 *μ*g/mL, respectively, as standard stock solutions. The calibration solutions were prepared by adding appropriate volumes of mixture working solution and 10 *μ*L of IS into 100 *μ*L blank rat plasma, resulting in concentrations: 70, 140, 350, 700, 1400, 3500, 7000, and 14000 ng/mL for safrole and 50, 100, 250, 500, 1000, 2500, 5000, and 10000 ng/mL for methyl eugenol. Quality control (QC) samples at three levels (low, medium, and high concentration) were prepared in the same manner. All the samples were kept at 4°C before use.

### 2.5. Preparation of Plasma Sample

100 *μ*L plasma sample was incorporated with 10 *μ*L n-hexane and 10 *μ*L of IS (5 *μ*g/mL) and vortexed for 30 seconds. 130 *μ*L *n*-hexane was then added to the sample and vortex-mixed for 5 min. The samples were centrifuged (12,000 × *g*, 10 min, 4°C), and 10 *μ*L supernatant was injected for GC-MS analysis.

### 2.6. Method Validation

#### 2.6.1. Specificity

The specificity was measured by comparing the chromatograms of blank plasma, blank plasma spiked with analytes and IS, and plasma samples obtained after oral administration of *Asarum* essential oil extracts.

#### 2.6.2. Linearity and Sensitivity

To assess the linearity, we constructed a calibration curve by analyzing the spiked calibration samples. The calibration curve was measured by 1/x weighted regression, and it was constructed based on peak area ratios of analytes against the internal standards. The lower limit of detection (LLOD) was defined as a concentration that S/N ratio of 3, and the lower limit of quantification (LLOQ) was determined as the concentration which provided a signal-to-noise (S/N) ratio of 10. The acceptable accuracy (RE) was within ±20%, and the relative standard deviation (RSD) was below 20%.

#### 2.6.3. Precision and Accuracy

The intra- and interday accuracy and precision each day and three consecutive days were evaluated by measuring quality control (QC) samples at three different concentration levels (120, 1200, and 12000 ng/mL for safrole; 80, 800, and 8000 ng/mL for methyl eugenol). The relative error (RE, %) and the relative standard deviation (RSD, %) were used for expressing the intra- and interday accuracy and precisions, respectively.

#### 2.6.4. Matrix Effect and Extraction Recovery

The low, medium, and high concentration samples in six replicates were used to analyze the extraction recovery. The extraction recovery was calculated through comparing the peak areas of QC samples with those in postextracted spiked samples. The matrix effects were studied by comparing the peak areas of QC samples spiked postextraction to those acquired from nonextracted samples.

#### 2.6.5. Stability

The stability was evaluated under different condition with variable time and temperature including long-term stability (storing the QC samples at -80°C for 7 days), short-term stability (storing the QC samples at room temperature for 4 h), freeze/thaw stability (three freeze (-20°C) thaw cycles), and auto-sampler stability (incubating the spiked samples for 12 h in auto-sampler).

### 2.7. Application

All animal protocols were approved by the Laboratory Animal Ethics Committee of Tianjin University Traditional Chinese Medicine (TCM-LAEC20190059). Six male Sprague–Dawley rats (body weight 240 ± 10 g) were fasted for 12 h prior to the study, with free access to water. The *Asarum* extracts were administered to rats orally at a dose of 20 g/kg. 0.5% CMC-Na aqueous solution was used to dissolve the *Asarum* extracts to a concentration of 2 g/mL suspension and stored at 4°C. Approximately 220 *μ*L blood samples were collected from the suborbital vein into centrifuge tubes at 0, 0.08, 0.17, 0.5, 1, 2, 4, 6, 8, 10, 12, 24, 36, 48, and 60 h postadministration in rats. The plasma was separated by centrifugation at 4000 × *g* for 10 min immediately and frozen at -80°C until the time of analysis. The main program pharmacokinetic parameters were processed by Drug and Statistics 3.0 (Medical College of Wannan, China).

## 3. Results and Discussion

### 3.1. Optimization of Pretreatment Procedure

For the pretreatment of plasma samples, two different pretreatment methods (liquid-liquid extraction and protein precipitation methods) were utilized in this study. Results showed that liquid–liquid extraction (LLE) with *n*-hexane and ethyl acetate both could obtain reproducible extraction and good recovery for the analytes, while the protein precipitation extraction with methanol and acetonitrile was found to contain endogenous interference. However, considering that the plasma endogenous substances interfere with the determination of the analytes after the ethyl acetate treatment, *n*-hexane was chosen to be the extraction solvent for this experiment.

### 3.2. Optimization of Internal Standard Compounds

In order to ensure the accuracy of the experimental determination, the relative molecular mass, boiling point, and retention time of the internal standard compound and the analytes should be similar and can be separated well. The experiment investigated the influence of menthone and m-xylene as internal standard compounds on the analysis samples. The results demonstrated that the menthone's structure was similar to the analytes, and it can be separated well within 4.5 min, which satisfied the measurement requirements. Therefore, menthone was used in the experiment as the internal standard.

### 3.3. Method Validation

#### 3.3.1. Specificity

**Figure 3:**
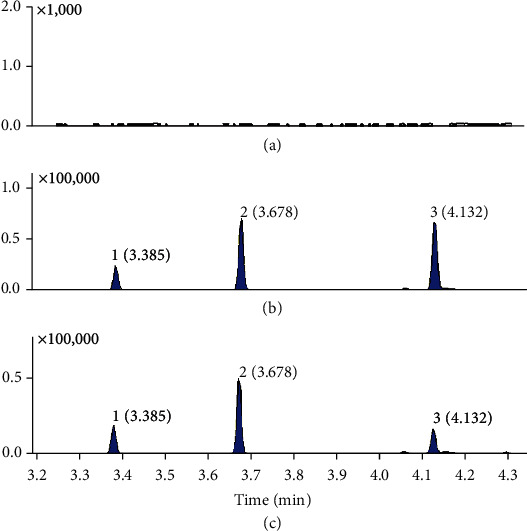
The typical chromatograms of (1) IS, (2) safrole, and (3) methyl eugenol: (a) blank plasma, (b) blank plasma spiked with analytes and IS, and (C) plasma sample.

The SIM chromatograms of blank plasma, plasma spiked with safrole, methyl eugenol and IS, and plasma sample obtained after oral administration *Asarum* extracts were shown in Figure 3. The retention time of safrole, methyl eugenol, and menthone were 3.678, 4.132, and 3.385 min, respectively. There were no interference peaks at the retention time of analytes and IS.

#### 3.3.2. Linearity and Sensitivity

The calibration curves, the linearity ranges, LLOQ, and LLOD values for the analytes are summarized in Table 1. The plasma calibration curves exhibited good linear relationship within the range of 70-14000 ng/mL for safrole and 50-10000 ng/mL for methyl eugenol. The correlation coefficients of the two compounds were both 0.999.

#### 3.3.3. Precision and Accuracy

The data of the intra- and interday precision and accuracy are summed up in Table 2. The results showed that at each QC level of two compounds, the RSD values were below 9.8% for intra- and interday precisions, and the RE values of average accuracy were from −7.9% to 11.4%.

#### 3.3.4. Matrix Effect and Extraction Recovery

The mean matrix effect ratio for all analytes and IS ranged from 80.6 ± 2.2% to 104.8 ± 7.4%, and the mean recovery rates of safrole and methyl eugenol ranged from 84.6 ± 5.6% to 97.6 ± 5.5% (Table 3). The results demonstrated that the influence from the plasma matrix was negligible.

#### 3.3.5. Stability

The stability of analytes in rat plasma under different conditions is summed up in Table 4, which shows that the RSD value of the replicate QC samples was less than 9.9%. Safrole and methyl eugenol in plasma samples were found to be stable under all conditions studied.

### 3.4. Pharmacokinetic Studies

**Figure 4:**
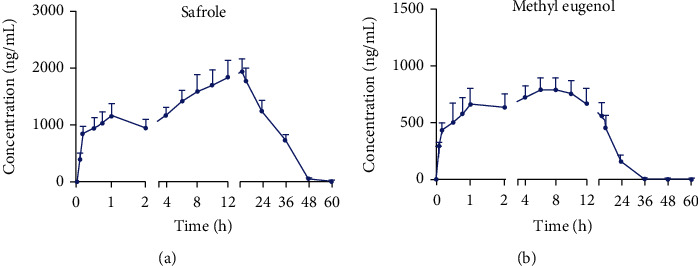
Mean plasma concentration–time curves of safrole and methyl eugenol after oral administration of Asarum extract (mean ± SD, *n* = 6).

The validated method in this paper was successfully applied to the pharmacokinetic study of safrole and methyl eugenol in rat plasma after oral administration of the essential oil extracts of *Asarum*.  Figure 4 shows the mean plasma concentration–time profile of the bioactive ingredients and the calculated main pharmacokinetic parameters is presented in Table 5.

The main pharmacokinetic parameters are listed in Table 5 including the maximum plasma concentration *C*_max_ and time to reach *C*_max_ of each compound (*T*_max_). The *T*_1/2_ was the elimination half-life, and it reflects the elimination rate of the drug in the body. The area under the plasma concentration-time curve AUC_(0 − *t*)_ was calculated using the trapezoidal rule and extrapolated to infinity for AUC_(0 − ∞)_. The mean residence time (MRT) of safrole and methyl eugenol was also investigated.

As seen from the results, the *C*_max_ of safrole and methyl eugenol was 1979.5 ± 267.1 and 823.0 ± 123.8, separately, indicating that the blood concentrations of the two compounds were high and that their absorptions were complete. The *T*_max_ of safrole and methyl eugenol was 13.00 ± 1.10 h and 6.67 ± 2.07 h, respectively, showing that the absorption of methyl eugenol was faster than safrole. The *T*_1/2_ of safrole and methyl eugenol was 2.44 ± 2.72 and 3.67 ± 1.60 h, respectively. The AUC_(0 − *t*)_ and the AUC_(0 − ∞)_ of both two compounds were close by, indicating that the monitoring time of this study was appropriate. The result of mean residence time was consistent with the concentration–time profile that the long mean residence time of safrole (18.39 ± 0.64 h) allowed it to be detectable in plasma samples after 48 h. Comparatively, methyl eugenol was completely metabolised after 36 hours in view of its short mean residence time (10.61 ± 0.63 h).

Previous research found that the pharmacokinetic profiles of the analytes in traditional Chinese medicine (TCM) were slightly different from that observed for their pure forms or other prescriptions [27]. It might due to the complexity of TCM for their interactions among so many compounds in vivo. Therefore, using *Asarum* extracts to study the pharmacokinetics of safrole and methyl eugenol can provide a more scientific explanation of the pharmacokinetic changes of these two components in vivo. This work can provide the experimental basis for the clinical use of *Asarum* and its prescriptions.

## 4. Conclusion

A rapid and sensitive GC-MS method with SIM mode was developed to determine safrole and methyl eugenol in rat plasma after oral administration of the essential oil extracts of *Asarum* in this study. It has been found that safrole and methyl eugenol both can be absorbed into blood well. Safrole was absorbed at a slower rate in vivo when compared to methyl eugenol. This is the first time that reporting a GC–MS method used for the accurate determination and pharmacokinetic analysis of safrole and methyl eugenol in rats plasma after oral administration of the essential oil of *Asarum* with given concentrations of 20 g/kg. The pharmacokinetic parameters provided valuable information for the further development and clinical application of *Asarum*.

## Figures and Tables

**Table 1:** Calibration curves, correlation coefficients, linear ranges LLOD, and LLOQ of the 2 analytes.

Compounds	Calibration curves	Correlation coefficients (*r*^2^)	Linear range (ng/mL)	LLOD (ng/mL)	LLOQ (ng/mL)
Safrole	*Y* = 1.13 *e*^−003^*X* − 3.34 *e* − 002	0.999	70–14000	10.5	35
Methyl eugenol	*Y* = 1.59 *e*^−003^*X* − 4.52 *e* − 002	0.999	50–10000	7.5	25

**Table 2:** Precision and accuracy of 2 analytes in rat plasma (*n* = 6).

Compounds	Spiked concentration (ng/mL)	Intraday			Interday		
		Measured concentration (ng/mL)	Accuracy (RE, %)	Precision (RSD, %)	Measured concentration (ng/mL)	Accuracy (RE, %)	Precision (RSD, %)
Safrole	120	131.1 ± 9.9	9.2	7.6	133.7 ± 8.2	11.4	6.1
	1200	1302.3 ± 14.2	8.5	1.1	1329.3 ± 28.7	10.8	2.2
	12000	12658.9 ± 83.5	5.5	0.7	12769.7 ± 185.2	6.4	1.5
Methyl eugenol	80	86.6 ± 8.5	8.2	9.8	75.6 ± 6.0	-5.5	8.0
	800	746.0 ± 13.8	-6.8	1.9	742.4 ± 14.4	-7.2	1.9
	8000	7365.0 ± 46.9	-7.9	0.6	7493.9 ± 172.4	-6.3	2.3

**Table 3:** Matrix effect and extraction recovery of 2 analytes (*n* = 6).

Compounds	Spiked concentration (ng/mL)	Extraction recovery (%)	RSD (%)	Matrix effect (%)	RSD (%)
Safrole	120	91.8 ± 2.6	2.8	80.6 ± 2.2	2.7
	1200	90.7 ± 3.6	4.0	90.9 ± 7.6	8.3
	12000	84.6 ± 5.6	6.6	88.0 ± 3.5	3.9
Methyl eugenol	80	92.5 ± 3.6	3.9	88.6 ± 3.5	3.9
	800	87.9 ± 4.4	5.0	97.6 ± 5.7	5.9
	8000	97.6 ± 5.5	5.6	104.8 ± 7.4	7.1

**Table 4:** Stability of 2 analytes in rat plasma (*n* = 6).

Compounds	Spiked concentration (ng/mL)	Room temperature for 4 h		Three freeze-thaw cycles		Auto-sampler for 12 h		-80°C for 7 days	
		Measured concentration (ng/mL)	RSD (%)	Measured concentration (ng/mL)	RSD (%)	Measured concentration (ng/mL)	RSD (%)	Measured concentration (ng/mL)	RSD (%)
Safrole	120	132.5 ± 12.4	9.3	137.0 ± 10.6	7.8	136.3 ± 11.8	8.7	135.1 ± 11.0	8.2
	1200	1306.7 ± 15.7	1.2	1319.7 ± 21.2	1.6	1271.1 ± 3.3	0.3	1348.1 ± 41.4	3.1
	12000	12751.7 ± 38.6	0.3	12526.3 ± 331.5	2.7	12065.2 ± 210.7	1.8	12741.7 ± 103.1	0.8
Methyl eugenol	80	89.6 ± 8.8	9.9	78.5 ± 6.5	8.2	73.9 ± 6.1	8.2	81.0 ± 5.2	6.5
	800	751.7 ± 7.8	1.0	708.5 ± 16.2	2.3	727.7 ± 6.0	0.8	749.8 ± 40.7	5.4
	8000	7418.6 ± 62.9	0.9	7287.0 ± 522.7	7.2	7100.8 ± 230.4	3.2	7367.9 ± 638.7	8.7

**Table 5:** Main pharmacokinetic parameters of 2 analytes in rat plasma (*n* = 6, mean ± SD).

Parameters	Safrole	Methyl eugenol
*C*_max_ (ng/mL)	1979.5 ± 267.1	823.0 ± 123.8
*T*_max_ (h)	13.00 ± 1.10	6.67 ± 2.07
*T*_1/2_ (h)	2.44 ± 2.72	3.67 ± 1.60
AUC_(0 − *t*)_ (h ng/mL)	52974.6 ± 5289.0	15226.2 ± 2761.3
AUC_(0 − ∞)_ (h ng/mL)	53006.3 ± 5264.2	15276.2 ± 2785.4
MRT_(0 − *t*)_ (h)	18.40 ± 0.64	10.61 ± 0.63
MRT_(0 − ∞)_ (h)	18.39 ± 0.63	10.61 ± 0.63

## Data Availability

The data used to support the findings of this study are available from the corresponding author upon request.
